# Influence of Cow Bone Particle Size Distribution on the Mechanical Properties of Cow Bone-Reinforced Polyester Composites

**DOI:** 10.1155/2013/725396

**Published:** 2013-11-14

**Authors:** Isiaka Oluwole Oladele, Temitope Akinyemi Adewole

**Affiliations:** Department of Metallurgical and Materials Engineering, Federal University of Technology, Akure, Nigeria

## Abstract

This work was carried out to investigate the influence of cow bone particle size distribution on the mechanical properties of polyester matrix composites in order to consider the suitability of the materials as biomaterials. Cow bone was procured from an abattoir, washed with water, and sun-dried for 4 weeks after which it was crushed with a sledge hammer and was further pulverized with laboratory ball mill. Sieve size analysis was carried out on the pulverized bone where it was sieved into three different sizes of 75, 106, and 300 μm sieve sizes. Composite materials were developed by casting them into tensile and flexural tests moulds using predetermined proportions of 2, 4, 6, and 8%. The samples after curing were striped from the moulds and were allowed to be further cured at room temperature for 3 weeks before tensile and flexural tests were performed on them. Both tensile and flexural strength were highly enhanced by 8 wt% from 75 μm while toughness was highly enhanced by 6 and 8 wt% from 300 μm. This shows that fine particles lead to improved strength while coarse particles lead to improved toughness. The results show that these materials are structurally compatible and are being developed from animal fibre based particle; it is expected to also aid the compatibility with the surface conditions as biomaterials.

## 1. Introduction

As in other areas of biomedical research, nature is seen in the area of biocomposites as a guide to design new materials [1]. Mimicking the solutions found in natural materials is one of the most promising ways to reach the target set of properties needed in implant materials. Ideally, a replacement material should mimic the living tissue from a mechanical, chemical, biological, and functional point of view. It is very difficult to combine all these properties in only one material. Generally, tissues are grouped into soft and hard tissues. Bone and tooth are examples of hard tissue whereas skin, blood vessels, and cartilage are examples of soft tissue. Accordingly, hard tissues are intended to support loads, being stiffer (higher elastic modulus) and stronger (higher tensile strength) than soft tissues. On the other hand unreinforced polymers are typically more ductile but are not stiff enough to be used to replace hard tissues in load-bearing applications. Nevertheless, polymer based composites can be designed to meet stiffness and strength requirements for hard tissue substitution [2].

Nowadays research in polymer science and technology is mainly focused on composites made from renewable resources [3]. Biocomposites from renewable resources gained much importance universally, because of their biodegradable nature. Biocomposites are the most suitable materials profound in nature for their use in various fields due to their ecofriendly advantages. Bio-composites are manufactured using biopolymer as binder and natural fibre as the reinforcement material [4].

A large number of polymer associated implantable devices are used in medicine today. Polymer based biomaterials such as bone plates, ligaments, intervertebral discs, heart valves, and pacemakers are used to replace or reestablish function of failing tissues or organs. These biomaterials help heal, increase function, repair abnormalities, and thus improve the patient's quality of life [5].

Polymers are utilized in numerous medical applications. This is mainly due to their versatility as their composition, properties, and forms can be manipulated to readily produce shapes and structures in the form of gels, films, fibers, and solids [5, 6].

Cow bones are readily available in Nigeria due to large number of cows that are being slaughtered daily to give meat. In most cases, the bones from these cows constitute waste and pollute the environment. This work was carried out to investigate the applicability of cow bone particles which is animal fibre to reinforce polyester that has been used both as matrix and fibre for biomaterials before to develop biomaterials with suitable mechanical properties. Cow bone being natural animal fibre is expected to have good surface compatibility in addition to the structural compatibility requirements as biomaterials. The effect of particle size distribution on the mechanical properties of the polyester composites was explored.

## 2. Materials and Methods

The main materials that were used for this work are as follows: unsaturated polyester resin, cow bone, methyl ethyl ketone peroxide (MEKP) used as the catalyst, cobalt 2% in solution used as the accelerator, polyvinyl acetate used as the mould releasing agent, and ethanol used as a cleaning agent.

### 2.1. Material Preparation

The cow bone was procured from the abattoir, washed with water so as to remove the dirty particles that might have been stuck to the bone, and sunudried for 4 weeks after which they were crushed with hammer and finally pulverized using Denver laboratory ball mill. The particles from the process were sieved with sieve shaker 16155 model into 75, 106, and 300 *μ*m sieve sizes.

### 2.2. Mould Production

Tensile mould of gauge length 90 × 5 × 3 mm of a dumb-bell shape and flexural mould of 150 × 50 × 3 mm were used for the production of tensile and flexural samples, respectively, from where the hardness samples were obtained. 

### 2.3. Production of Composites

To develop the composites, 1.5 g each of catalyst and accelerator was added to 120 g of the polyester resin while bone particulate was varied in a predetermined proportion of 2, 4, 6, and 8 wt%. After proper stirring, the homogenous slurry is poured into the mould and allowed to be cured at room temperature before it is removed. Three (3) samples were produced for each mechanical property that was carried out from each proportion. The striped samples are left to be cured further at room temperature for 3 weeks before the mechanical tests were carried out.

### 2.4. Mechanical Testing and Structural Characterization of Cast Samples

Following the moulding of the composites, samples were prepared for tensile, flexural, and hardness tests. Scanning electron microscope (SEM) was used to investigate the miscibility between the fibre and matrix at the fractured surfaces. These tests were carried out as follows.

(a) *Determination of the Tensile Properties of the Materials.* In the present study, tensile tests were performed on INSTRON 1195 at a fixed crosshead speed of 10 mm min^−1^. Samples were prepared according to ASTM D412 (ASTM D412 1983) and tensile strength of the standard and conditioned samples was calculated.

(b) *Determination of the Flexural Property of the Materials.* Flexural test was carried out by using Testometric Universal Testing Machine in accordance with ASTM D790. To carry out the test, the grip for the test was fixed on the machine, the sample that has been cut into the test piece dimensions of 150 mm × 50 mm × 3 mm was hooked on the grip, and the test commenced. As the specimen is stretched, the computer generates the required data and graphs. The flexural test was performed at the speed of 100 mm/min.

(c) *Determination of the Hardness Property of the Materials.* Hardness test was carried out in accordance with ISO R 868, using shore D. The test was carried out by impressing the sample with the tip of the indenter for five seconds before taken the readings from the calibrated scale. Ten readings were taking for each sample and the average value was used as the representative value for the mechanical tests carried out.

(d) *SEM Observation.* SEM of the composites was observed using Zeiss SEM: Zeiss Ultra Plus 55 FECSEM, Zeiss, Oberkochen, Germany. Before the examination, the samples were prepared by cutting them with bench vice and hacksaw followed by gluing on sample holder and finally coated with carbon using Carbon Coater: EMITECH K950X, EM Technologies, Kent, England.

## 3. Results and Discussions

### 3.1. Variation of Tensile Properties with Fibre Content

**Figure 1:**
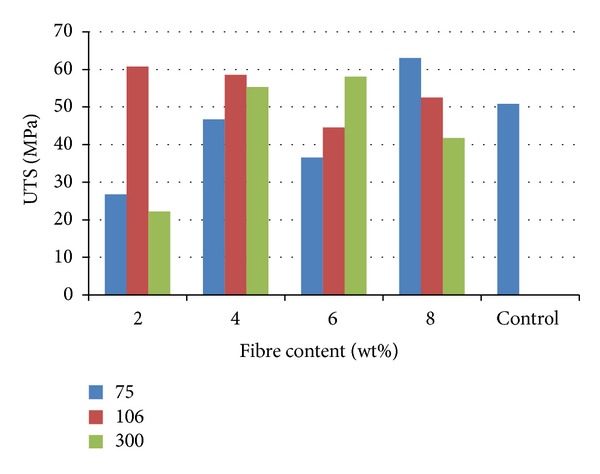
Ultimate tensile strength of cow bone-reinforced polyester composites.

Figure 1 shows the variation of ultimate tensile strength with the fibre content for the cow bone particulate reinforced polyester composites. It was observed from the result that sample reinforced with 8 wt% from 75 *μ*m particulate enhanced the tensile strength of the polyester matrix more than others. The strength was 63.04 MPa compared to the unreinforced polyester matrix that has a value of 50.76 MPa. This is likely due to large surface area that is possible from fine particles compared to large ones. 

**Figure 2:**
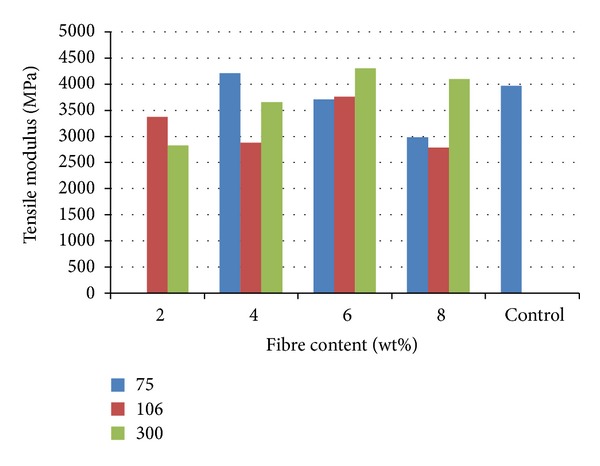
Tensile modulus of cow bone-reinforced polyester composites.

Variation of tensile modulus with fibre content is shown in Figure 2 where it was observed that 6 wt% from 300 *μ*m particulate reinforced polyester composite gave the best result. The value was 4304.7 MPa compared to unreinforced polyester matrix with a value of 3966.15 MPa. The tensile modulus is a parameter that measures the stiffness of the material. The result shows that the tensile modulus increases as the fibre content increases from 2 to 6 wt% for 300 *μ*m particulate reinforcement while it decreases for 8 wt% reinforced composite. This trend was also observed in Figure 1 for UTS which shows that the tensile properties of the composites increases as the fibre content increases from 2 to 6 wt% before it experiences depreciation with 300 *μ*m particulate reinforcement. 

The results from Figures 1 and 2 show that 6 wt% of 300 *μ*m and 8 wt% of 75 *μ*m reinforcement gave the best tensile properties compared to other samples. The tensile strength values obtained from these samples fall within the range of values stated by Williams [7]. Generally, in biomaterials, high strength of excess value is undesirable as this produces adverse bone remodeling and stress shielding, which over the long term leads to reduction in bone mass and implant loosening, especially in the proximal region. Fibre composites can be tailored to match the specific mechanical properties of the adjacent bone [8, 9]. This is done in order to avoid stress accumulation and system damage.

### 3.2. Variation of Flexural Properties with Fibre Content

**Figure 3:**
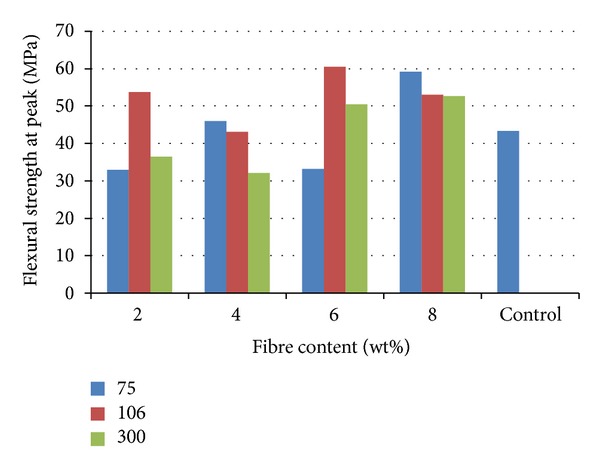
Flexural strength at peak of cow bone-reinforced polyester composites.

Figure 3 shows the result of the flexural strength at peak for the various samples. From the result, it was observed that flexural strength at peak was better enhanced by the addition of bone particles. However, the best reinforcement was achieved when 6 wt% from 106 *μ*m particle was added. The value was 60.42 MPa which was closely followed by 8 wt% from 75 *μ*m particulate reinforcement with a value of 59.14 MPa while the value for the unreinforced polyester was 43.25 MPa. Flexural property is a parameter that measures the ability of the material to resist deformation under bending stress.

**Figure 4:**
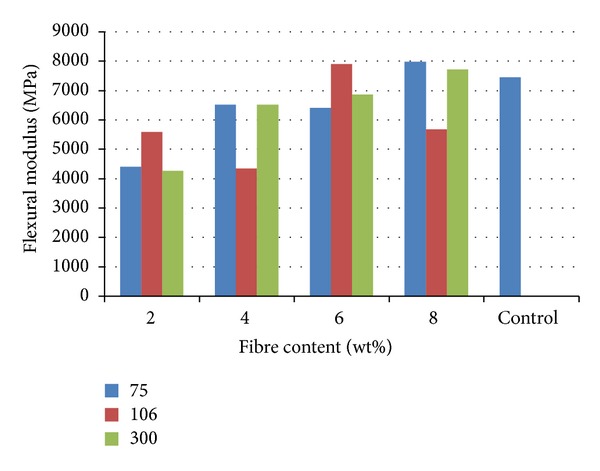
Flexural modulus of cow bone-reinforced polyester composites.

Figure 4 shows the variation of flexural modulus with fibre content. The result shows that 8 wt% from 75 *μ*m particulate reinforcement with a value of 7971.1 Mpa was closely followed by 6 wt% from 106 and 8 wt% from 300 *μ*m particles with values 7889.8 and 7709.5 MPa reinforcement respectively. These samples possess better flexural modulus compared to unreinforced polyester matrix with a value of 7451.8 MPa. The result shows that 300 *μ*m particle reinforcement increases the flexural modulus as the fibre content increases from 2 to 8 wt%.

### 3.3. Variation of Hardness Properties with Fibre Content

**Figure 5:**
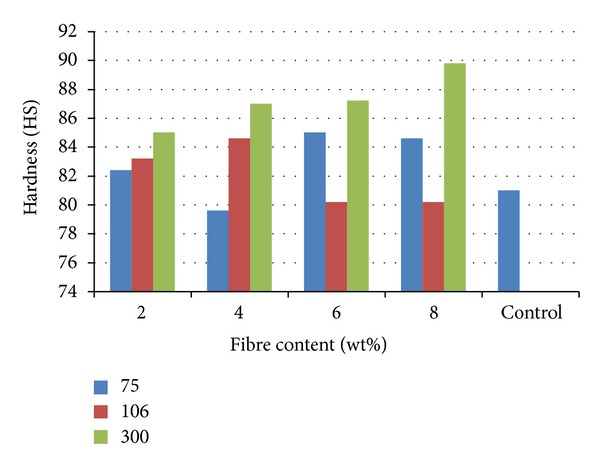
Hardness of cow bone-reinforced polyester composites.

Hardness property is a measure of the resistance of the materials to surface indentation and wear.  Figure 5 shows the variation of this property with the samples from where it was noticed that the reinforcement leads to the enhancement of the hardness property in almost all the samples produced. The best result was obtained for 8 wt% from 300 *μ*m reinforced sample with a value of 89.8 HS compared to the unreinforced polyester matrix with a value of 81 HS. The result shows that 300 *μ*m particle reinforcement increases the hardness as the fibre content increases from 2 to 8 wt%. This trend was similar to that of the flexural modulus in Figure 4.

### 3.4. SEM Micrographs of the Particulate Reinforced Polyester Composites

**Figure 6:**
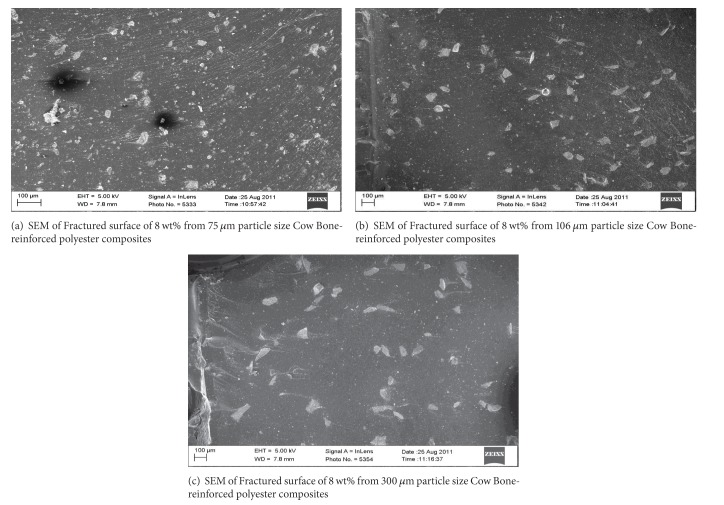
(a)–(c) show the SEM micrograph of cow bone particulate reinforced polyester composites.

Figure 6 depicts the SEM micrographs of the particulate reinforced polyester composites (Figures 6(a), 6(b), and 6(c)). From the micrographs, it was observed that there is proper dispersal of the bone particles (white particles) in the polyester matrix (black surface).

Figure 6(a) revealed more dispersal of the 75 *μ*m particle compared to others. This was due to the finest of the particle that lead to better mechanical properties. From Figures 6(a), 6(b), and 6(c), it was observed, that as the particle size increases, the number of particles that are present decreases due to weight increase. The micrographs revealed that there is proper bonding between the bone particles and the polyester matrix which was responsible for the good mechanical properties that was obtained from the mechanical tests results. As a result of the good wettability between the fibre and the matrix as well as adequate particle dispersal in the polyester matrix, better enhancement of properties was obtained for the composites in all compared to the unreinforced polyester matrix.

## 4. Conclusion

The use of animals as a means of testing for the suitability of some drugs in the area of drug delivery in human body has initiate the idea of using cow bone as a reinforcement in polyester in order to develop polymer based composites for biomedical application in this research. As a result of the compatibility between animals and human being, both being living things and in the class of animals, the use of cow bone as a replacement in human body is a welcome development. From this work, was observed the following.

- The use of cow bone particles 75,106 and 300 *μ*m led to the enhancement of the mechanical properties of polyester matrix. Polyester from previous research has been used as biomaterial which shows that it is a good material for this application in terms of compatibility. Though biocompatibilization treatment was not performed on the cow bone, this can be carried out on cow bone since it has met the mechanical properties necessary for the material to be used as biomaterial.
- In most of the results, 6 and 8 wt% from 75 and 300 *μ*m emerge as the best. This was the case as follow; UTS, 8 wt% from 75 *μ*m while for tensile modulus 6 wt% from 300 *μ*m. For flexural properties, 8 wt% from 75 *μ*m while for hardness, it was 8 wt% from 300 *μ*m.
- Both tensile and flexural strength were highly enhanced by 8 wt% from 75 *μ*m while toughness was highly enhanced by 6 and 8 wt% from 300 *μ*m. This shows that fine particles lead to improved strength while coarse particles lead to improved toughness.
